# Agonist Antagonist Interactions at the Rapidly Desensitizing P2X3 Receptor

**DOI:** 10.1371/journal.pone.0079213

**Published:** 2013-11-01

**Authors:** Nick Helms, Maria Kowalski, Peter Illes, Thomas Riedel

**Affiliations:** Rudolf Boehm Institute for Pharmacology and Toxicology, University of Leipzig, Leipzig, Germany; Dalhousie University, Canada

## Abstract

P2X3 receptors (P2XRs), as members of the purine receptor family, are deeply involved in chronic pain sensation and therefore, specific, competitive antagonists are of great interest for perspective pain management. Heretofore, Schild plot analysis has been commonly used for studying the interaction of competitive antagonists and the corresponding receptor. Unfortunately, the steady-state between antagonist and agonist, as a precondition for this kind of analysis, cannot be reached at fast desensitizing receptors like P2X3R making Schild plot analysis inappropriate. The aim of this study was to establish a new method to analyze the interaction of antagonists with their binding sites at the rapidly desensitizing human P2X3R. The patch-clamp technique was used to investigate the structurally divergent, preferential antagonists A317491, TNP-ATP and PPADS. The P2X1,3-selective α,β-methylene ATP (α,β-meATP) was used as an agonist to induce current responses at the wild-type (wt) P2X3R and several agonist binding site mutants. Afterwards a Markov model combining sequential transitions of the receptor from the closed to the open and desensitized mode in the presence or absence of associated antagonist molecules was developed according to the measured data. The P2X3R-induced currents could be fitted correctly with the help of this Markov model allowing identification of amino acids within the binding site which are important for antagonist binding. In conclusion, Markov models are suitable to simulate agonist antagonist interactions at fast desensitizing receptors such as the P2X3R. Among the antagonists investigated, TNP-ATP and A317491 acted in a competitive manner, while PPADS was identified as a (pseudo)irreversible blocker.

## Introduction

Besides the Cys-loop and glutamate receptor families, P2XRs comprise the third group of ligand-gated cation channels, consisting of seven subunits referred to as P2X1 through P2X7 [1,2]. They possess a large extracellular loop, two transmembrane domains and intracellular N- and C-termini [3]. Three homomeric or heteromeric P2XR subunits assemble into an ATP-activated ion channel by forming a central pore [5]. Although the sequence identity between the individual subtypes of P2XRs is rather high, the biophysical properties and agonist/antagonist sensitivities allow a rough classification into two large subgroups [4,6]. P2X1 and P2X3 homomeric receptors rapidly desensitize in the presence of ATP, whereas the other P2XR-types desensitize at a much slower rate. In addition, α,β-methylene ATP (α,β-meATP) is a highly selective agonist for P2X1 and P2X3, with practically no activity at P2X2,4-7. 

The particularly great importance of homomeric P2X3 and heteromeric P2X2/3Rs is given by their almost exclusive association with pain pathways in the organism [7,8]. These receptors were cloned from rat dorsal root ganglia (DRG) (P2X3 [9],; P2X2/3 [10],). The receptors situated on the peripheral terminals of DRGs react to ATP released by painful tissue damage or distension. The ensuing local depolarization triggers action potentials that are conducted *via* the DRG central terminals to the spinal cord dorsal horn [11]. In animal models, P2X3R antagonists and antisense oligonucleotides inhibit various acute and chronic pain states which arise e.g. during inflammation, neuropathy, migraine, and cancer [12,13]. Accordingly, P2X3R-deficient mice exhibit decreased nociceptive behaviour in comparison with their wild-type backgrounds in experimental pain states. Thus, the development of selective and reversible (competitive) P2X3 and P2X2/3 antagonists as therapeutic agents is an imminent challenge for pharmacologists/clinicians. 

The most direct method to investigate P2X3R-function is the measurement of the transmembrane current induced by agonist application. However, the evaluation of such measurements is difficult, because agonist binding and receptor activation (within the range of milliseconds) is counteracted by the slower but partly overlapping desensitization (within the range of seconds). In addition, the recovery from desensitization is still a slower process lasting for several minutes. Hence, the strongly desensitizing behaviour of P2X3Rs prevents a classic analysis of agonist-antagonist interaction by the usual Lineweaver-Burk or Schild plots. To circumvent this problem, the slowly desensitizing P2X2/3 or chimeric P2X2-3Rs were expressed in stable cell lines for testing P2X3R antagonist effects ([14,15]. The heteromeric P2X2/3R is composed of 1 P2X2 and 2 P2X3 subunits and therefore its agonist binding site is similar but not identical with that of the homomeric P2X3R [15]. In the chimeric P2X2-3R, the N-terminus and the adjacent first transmembrane domain of P2X3 is replaced by the analogous portion of P2X2; thereby the receptor desensitizes slowly although its agonist binding site is purely P2X3 [14]. 

Our experimental approach was different from the above ones. We extended a previously developed Markov model for agonist binding [16] with further parameters to model also antagonist binding. Eventually, a minimum number of two parameters (the association and dissociation rates of antagonists) were sufficient to simulate a variety of experimental conditions, such as the concentration-dependence of inhibition and the wash-in and wash-out kinetics. Additionally, we were able to correctly describe the modified current kinetics in the presence of an antagonist and the dynamic interaction of agonists and antagonists.

The mentioned Markov model was used to analyse the binding of the antagonists TNP-ATP, A317491, and PPADS to the wild-type (wt) P2X3R and to some of its binding site mutants, where individual amino acids (AAs) were replaced by alanine. We demonstrated that TNP-ATP and A317491 are rapidly reversible, competitive antagonists, whereas the effects of PPADS are *quasi* irreversible. It has also been shown that TNP-ATP and A317491 interact with some AAs within the agonist binding pocket which are important for binding the natural agonist ATP and its structural analogue α,β-meATP.

## Materials and Methods

### Cell Culture and Mutagenesis

HEK293 cells were kept in Dulbecco's modified Eagle medium (Sigma-Aldrich, St. Louis, MO) with 4.5 mg/ml glucose, 1% L-glutamine and 10% fetal calf serum, at 37°C, in humidified air (with 5% CO_2_). The human (h)P2X3R cDNA was subcloned into pIRES2-EGFP vector (Clontech Laboratories, Mountain View, CA) by using PstI and EcoRI restriction sites. All P2X3R mutants had been generated by introducing replacement mutations with the QuikChange site-directed mutagenesis protocol (Agilent Technologies, Santa Clara, CA). Individual AA residues located at one of the four nucleotide-binding segments of the P2X3R were replaced with alanin [17]. Before transfection, the cells were plated in plastic dishes. 0.5 µg of the receptor plasmid, 100 µl OptiMEM and 10 µl of PolyFect transfection reagent (QIAGEN, Valencia, CA) were incubated for 10 minutes and afterwards applied to the dishes. To remove residual plasmids the medium was replaced with OptiMEM after 18 h of incubation.

### Kinetic Fit of P2X3 Current with Hidden Markov Model

On the basis of a recently published Markov model, which describes the behaviour of P2X3R-channels during agonist binding [16], we created an extended model also accounting for antagonist actions. In the present extended model, we supposed that the binding of a competitive antagonist is just an alternative step to the binding of an agonist, and has no further consequences for the receptor, except to prevent agonist binding. We took account of this assumption by introducing 3 binding sites, 1 for each subunit, and presumed that they are occupied independently from each other. On this basis, the model becomes relatively basic, because there are only 2 new free parameters needed to describe the interaction of the antagonists with the receptor and the agonist.

**Figure 1:**
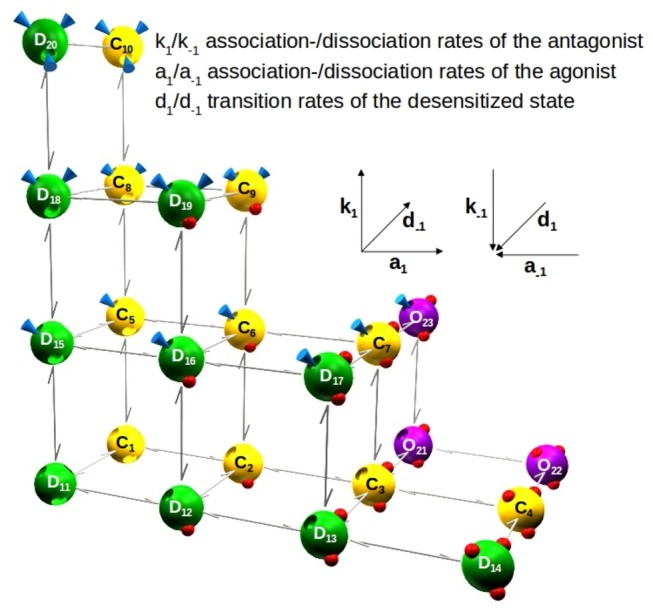
The Markov model for competitive antagonism consists of 3 different receptor states, closed (C; yellow), open (O; purple) and desensitized (D; green), which are connected by the specific transition rates for each state. Because every state can bind up to 3 ligands, which are either agonists (red spheres) or antagonists (blue cones), there are 23 states in this model. Starting at C1, an additional agonist is bound rightwards and an additional antagonist upwards. Contrary to this, the unbinding of agonists and antagonists proceeds in opposite directions. k_1_, k_-1_, association and dissociation rates of the antagonist; a_1_, a_-1_, association and dissociation rates of the agonist; d_1_, d_-1_, transition rates of the desensitized state. Insets: structures of the antagonists used in this study (Tocris).

P2X3Rs have 3 binding sites, and each one can be vacant, agonist-bound or antagonist-bound (Figure 1). This allows 10 possible combinations for the occupancy of the 3 binding sites; therefore, the model has 10 closed, and 10 desensitized states. In contrast, the model has only 3 open states, because at least two agonist molecules have to be bound to induce opening. Agonist and antagonist association and dissociation rates were calculated stoichiometrically, i.e. rate constants were multiplied by the number of available binding sites (see Table S1.)

In the scheme shown in Figure 1, agonist association and dissociation steps are plotted along the horizontal axis, while antagonist association and dissociation steps take place along the vertical axis. The receptor may transit from both closed and open states to the desensitized state. In order to reduce the number of free parameters in the model, several constraints have been added to tie certain rates. Thus, if one of the rates changes, all tied rates will change as well. The corresponding rates of the agonist depending on the alanin-mutants used, have been investigated previously and could be fixed accordingly [16]. Due to this approach, eventually only two free rates will remain in our model - the association and dissociation rates of the antagonist. 

### Calculation of the Dissociation Constant and Binding Energy; Data Analysis

Kinetic fits for the P2X3 current were calculated with the Mac-modul of the QuB software [18]. The dissociation constant *K*_*D*_ and the binding energy *ΔG* for receptor antagonist combination were calculated from the fit parameters *k*_*1*_ and *k*_*-1*_ of the Markov model with the equations *K*_*D*_= *k-1*/*k*_*1*_ and *ΔG*=*RTln K*_*D*_, where *R* is the gas constant and *T* is the absolute temperature. The S.D. values for the K_D_ values and binding energies were obtained from the propagated S.D. values for k_1_ and k_-1_ in the kinetic fits.

The concentration-inhibition curve for PPADS was fitted by using a three parametric Hill plot (OriginPro 8; Origin Lab Corp., Northampton, MA). The IC_50_ value was taken from the plot and is presented as mean±S.E.M. of n experiments. 

One way analysis of variance followed by the Holm-Sidak *post hoc* test was used for statistical analysis. A probability level of 0.05 or less was considered to reflect a statistically significant difference.

### Electrophysiological Studies

Whole-cell patch-clamp recordings were performed 2 to 4 days after transient transfection of the HEK293 cells at room temperature (20-25°C) by using an Axopatch 200B patch-clamp amplifier (Molecular Devices, Sunnyvale, CA). The pipette solution contained (in mM) CsCl 135, CaCl_2_ 1, MgCl_2_ 2, HEPES 20, EGTA 11, and GTP 0.3 (Sigma-Aldrich); the pH was adjusted to 7.3 with CsOH. The external physiological solution contained (in mM) KCl 5, NaCl 135, MgCl_2_ 2, CaCl_2_ 2, HEPES 10 and glucose 11; the pH was adjusted to 7.4 with NaOH. The pipette resistance ranged from 3 to 7 MΩ, the membrane resistance was 0.1 to 2 GΩ and the access resistance was 3 to 15 MΩ. All recordings were performed at a holding potential of -65 mV. Data were filtered at 1 kHz with the inbuilt filter of the amplifier, digitized at 2 kHz and recorded by using a Digidata 1440 interface and pClamp10.2 software (Molecular Devices). Access resistance was compensated mathematically as described before [16].

Drugs were dissolved in external solution and superfused to single cells by using a rapid solution-exchange system (SF-77B Perfusion Fast Step, Warner Instruments, Hamden, CT). To estimate the solution exchange times of the system KCl (150 mM) was applied to the cell and the resulting current was recorded. The time constant of solution-exchange was determined with a single exponential fit. This time constant was used to simulate the wash-in and wash-out of the solutions during the Markov fits. Between drug applications, the cells were continuously superfused with the standard external solution. In order to resolve the antagonist binding within the complex P2X3 kinetics it was necessary to design various application protocols. These protocols take account of the problems arising from e.g. slow association of the antagonist with the receptor and slow dissociation from it, distorted by desensitization, or rapid association with the receptor and rapid dissociation from it, distorted by the limited speed of the solution exchange, which is slower than the activation process. We used as an agonist the P2X1,3R-selective α,β-methylene ATP (α,β-meATP) throughout, in all series of experiments.

**Figure 2:**
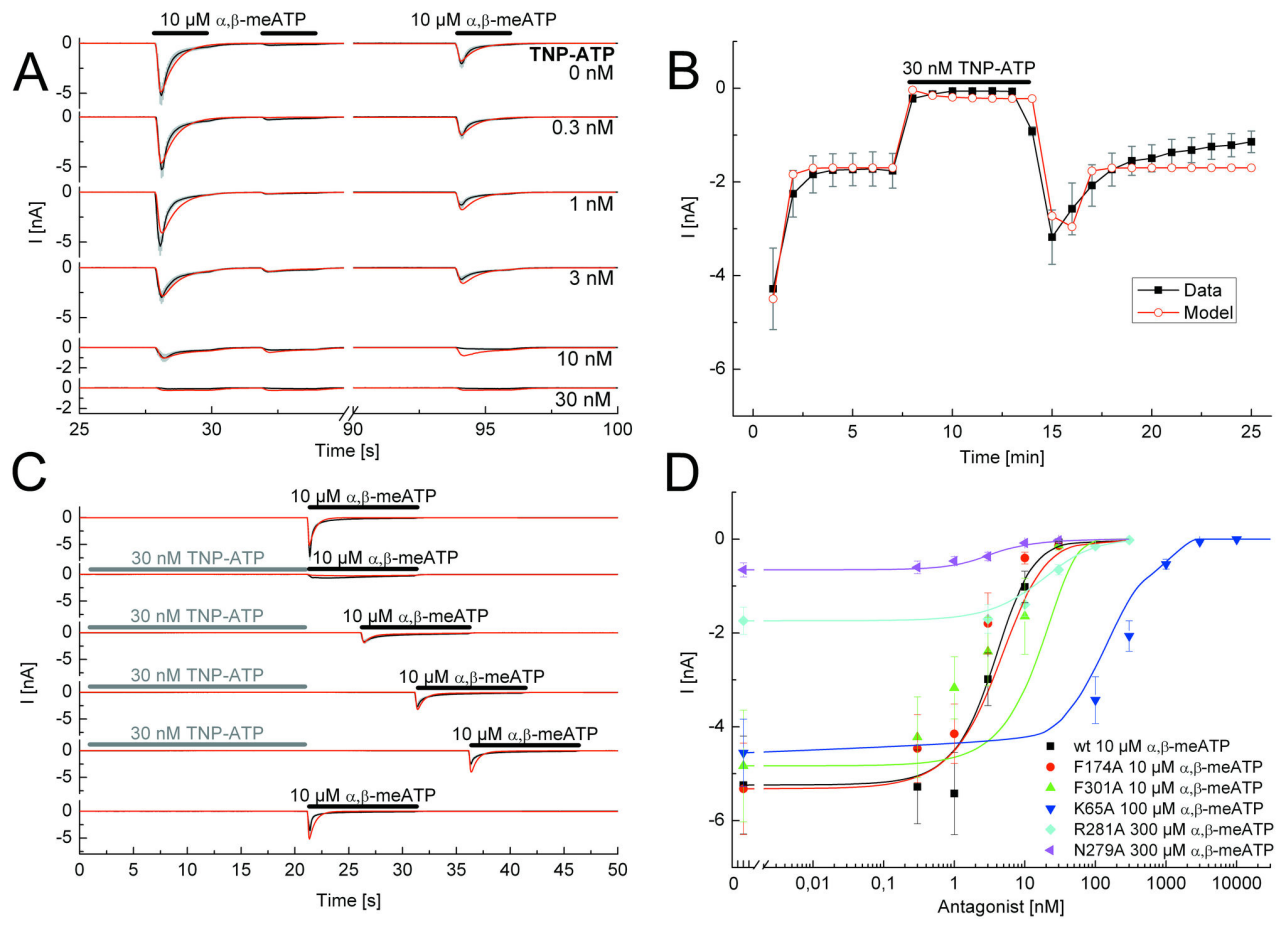
Application protocols used to investigate the nature of antagonism between TNP-ATP and α,β-meATP at the wild-type (wt) P2X3R and its binding site mutants. A, Steady-state application protocol for the wt P2X3R. α,β-meATP (10 µM) was superfused three times for 2 s each, with 2-s and 60-s intervals between subsequent applications, both in the absence and in the presence of increasing concentrations of TNP-ATP (0.3-30 nM; each agonist application cycle was spaced apart by 5 min). B, Dynamic antagonist application protocol. α,β-meATP (10 µM) was repetitively applied for 1 s each at an interval of 1 min. The onset and offset of the blockade by TNP-ATP (30 nM; 5 min) is shown. C, Wash-out protocol for the wt P2X3R. α,β-meATP (10 µM) application of 10-s duration was done either in the absence of TNP-ATP (30 nM) or at variable time-periods (up to 15 s, as indicated) after its wash-out; TNP-ATP was superfused for 25 s with 5 min intervals between each run. D, Concentration response-curves for the indicated mutant receptors simulated by the Markov model (lines) to fit the experimentally determined mean current amplitudes (symbols) without and with increasing concentrations of TNP-ATP (0.3 nM - 10 µM) in the superfusion medium. The F301A curve is misplaced with respect to the symbols. One possible explanation for this finding is that the simulation takes the kinetics, the association and dissociation rates and the recovery time into account and not only the amplitudes. α,β-meATP concentrations were adjusted for the requirements of every mutant. The black lines represent the experimentally measured P2X3R currents (A, C) or the lines connecting the experimentally determined mean values (B), with the grey bars as their S.E.M. The fitted currents have a red colour. Means ± S.E.M. of the data together with the generated concentration-response curves are shown in colour (D). The number of similar experiments for each group of data varied from 6-13. The thick horizontal lines above the current traces designate the duration of agonist or antagonist superfusion.

**Figure 3:**
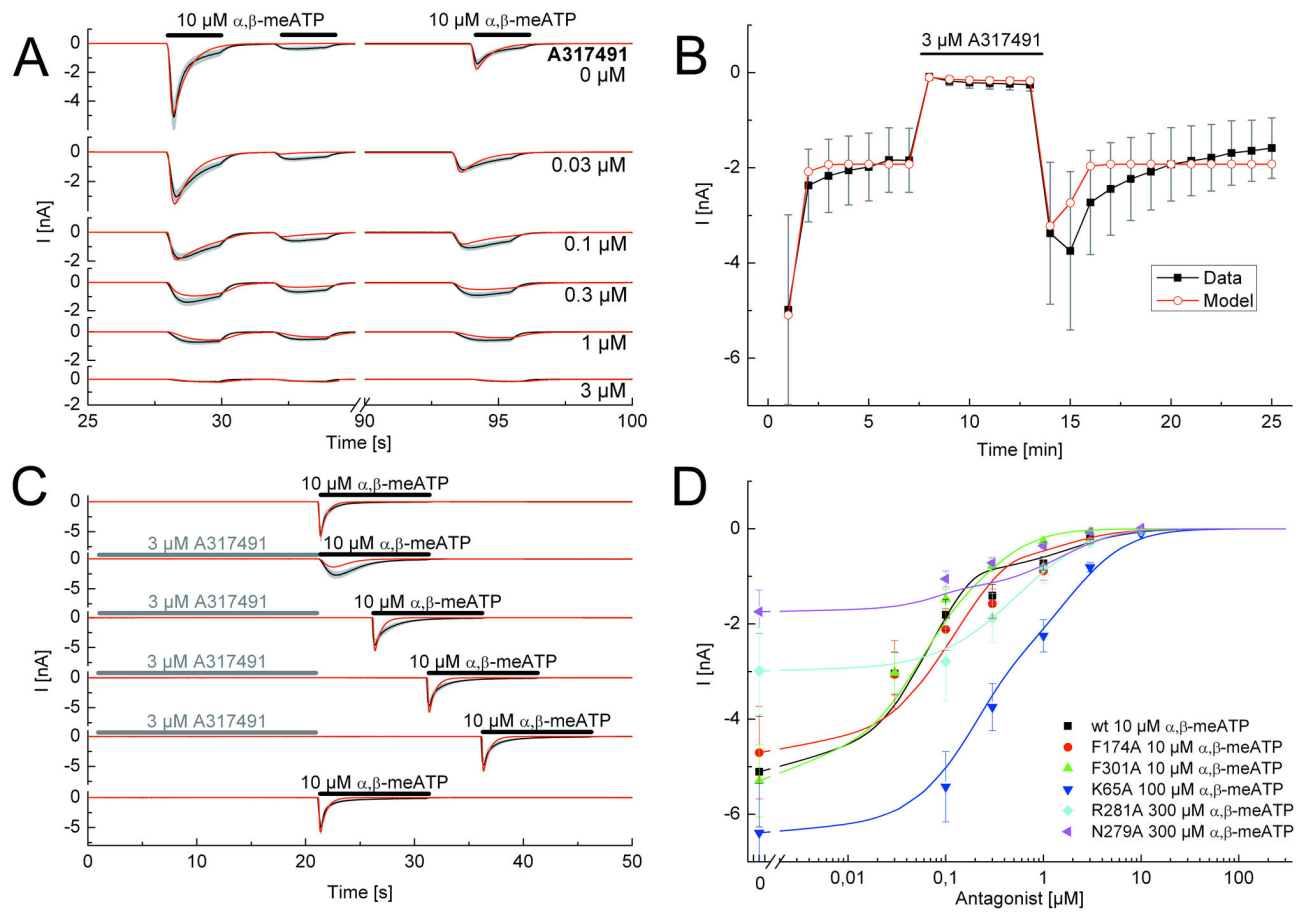
Application protocols used to investigate the nature of antagonism between A317491 and α,β-meATP at the wild-type (wt) P2X3R and its binding site mutants. A, Steady-state application protocol for the wt P2X3R. α,β-meATP (10 µM) was superfused three times for 2 s each, with 2-s and 60-s intervals between subsequent applications, both in the absence and in the presence of increasing concentrations of A317491 (0.03-3 µM; each agonist application cycle was spaced apart by 5 min). B, Dynamic antagonist application protocol. α,β-meATP (10 µM) was repetitively applied for 1 s each at an interval of 1 min. The onset and offset of the blockade by A317491 (3 µM; 5 min) is shown. C, Wash-out protocol for the wt P2X3R. α,β-meATP (10 µM) application of 10-s duration was done either in the absence of TNP-ATP (30 nM) or immediately after its wash-out; A317491 was superfused for 25 s with 5 min intervals between each run. D, Concentration response-curves for the indicated mutant receptors simulated by the Markov model (lines) to fit the experimentally determined mean current amplitudes (symbols) without and with increasing concentrations of A317491 (0.03-10 µM) in the superfusion medium. α,β-meATP concentrations were adjusted for the requirements of every mutant. The black lines represent the experimentally measured P2X3R currents (A, C) or the lines connecting the experimentally determined mean values (B), with the grey bars as their S.E.M.. The fitted currents have a red colour. Means ± S.E.M. of the data together with the generated concentration-response curves are shown in colour (D). The number of similar experiments for each group of data varied from 8-13. The thick horizontal lines above the current traces designate the duration of agonist or antagonist superfusion.

**Figure 4:**
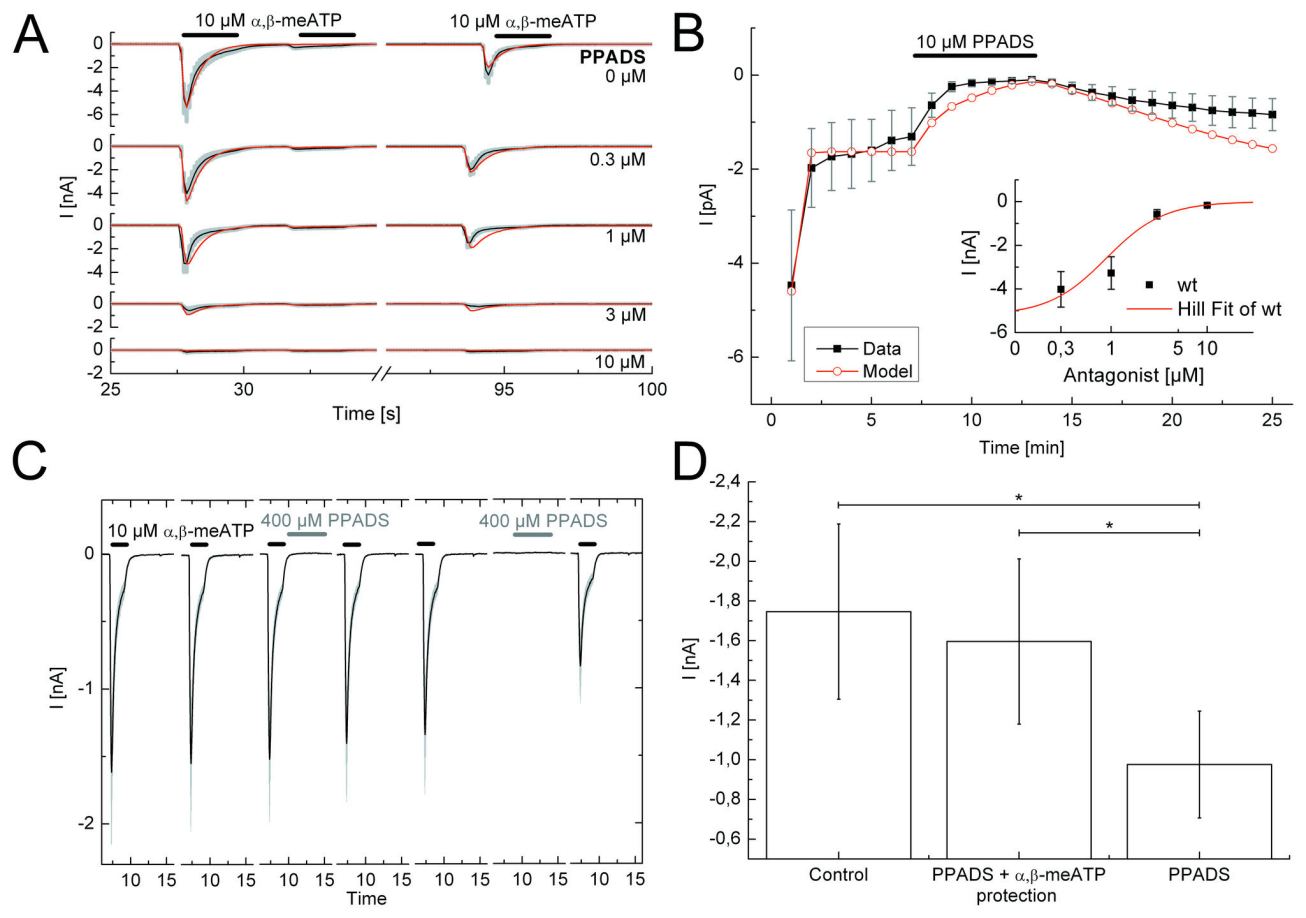
Application protocols used to investigate the nature of antagonism between PPADS and α,β-meATP at the wild-type (wt) P2X3R and its binding site mutants. A, Steady-state application protocol for the wt P2X3R. α,β-meATP (10 µM) was superfused three times for 2 s each, with 2 s and 60 s intervals between subsequent applications, both in the absence and in the presence of increasing concentrations of PPADS (0.03-10 µM; each agonist application cycle was spaced apart by 5 min). B, Dynamic antagonist application protocol. α,β-meATP (10 µM) was repetitively applied for 1 s each at an interval of 1 min. The onset and offset of the blockade by PPADS (10 µM; 5 min) is shown. C, Protection protocol for the wt P2X3R. Drug application was 7-times (S_1_-S_7_) with intervals of 5 min. α,β-meATP (10 µM) was applied for 2 s at S_1_-S_5_ and S_7_. Immediately after S_3_ and S_6_ (in this latter case without applying α,β-meATP), PPADS (400 µM) was superfused for 5 s. D, Summary of experiments shown in C. The PPADS-induced blockade of P2X3Rs is prevented by applying immediately before PPADS α,β-meATP. The black lines represent the experimentally measured P2X3R currents (A, C) or the lines connecting the experimentally determined mean values (B), with the grey bars as their S.E.M.. The number of similar experiments for each group of data varied from 7-9. The thick horizontal lines above the current traces designate the duration of agonist or antagonist superfusion. *P<0.05; statistically significant differences between the indicated columns.

The antagonist application protocols were the following:

(1) *Steady* *state* *protocol* (e.g. Figure 2A). In this protocol, we combined the construction of a concentration-response curve for the antagonist and the measurement of receptor kinetics (recovery from desensitization; [16]) by repetitively applying the agonist. In every run with increasing antagonist concentrations, the same concentration of the agonist was applied (2-s duration), 28 s, 32 s and 94 s after starting antagonist superfusion. After 5 minutes, which is sufficient for P2X3R to recover from desensitization, the next run with an increasing antagonist concentration was started. This protocol provides information about the concentration-inhibition relationship for antagonists, but gives no information about the kinetics of their receptor association and -dissociation.

(2) *Wash-out* *protocol* (e.g. Figure 2C). The steady-state protocol was combined with the wash-out protocol, when cells have been exposed for 20 s to a high antagonist concentration causing a complete block of the agonist induced current. Immediately after the antagonist application had been stopped, the agonist was applied for 10 s, which allowed a direct observation of the antagonist dissociation kinetics for fast unbinding antagonists. Then, we inserted increasing time intervals between antagonist and agonist application in order to follow the unbinding process. The interval between two runs was set to 5 min also.
(3) *Dynamic* *antagonist* *application* *protocol* (e.g. Figure 3B). For antagonists, whose maximum effect develops only at a minute time scale, we used a protocol that allows the observation of the dynamic replacement of the agonist by the antagonist and *vice* *versa*. The agonist was applied 25-times for 1 s each at an interval of 1 min. This period of time is too short for all receptors to recover from desensitization, but increases the frequency of time-points where the receptor responsivity can be observed. After the first 3 agonist applications, an equilibrium is achieved between receptors that are still desensitized and receptors that can already be activated. The 8th to 13th of 25 agonist applications occur in the presence of an antagonist.

(4) *Protection* *protocol* (e.g. Figure 4C). In order to find out whether the antagonist interacts in a competitive manner with the agonist, a protection protocol was used. In this protocol there are 7 time-points (S_1_-S_7_) with an interval of 5 minutes between each. The agonist was applied for 2 s at S_1_-S_5_ and S_7_. Immediately after S_3_ and S_6_ (in this latter case without a preceding agonist application) a stable antagonist concentration was superfused. If the antagonist occupies the same site as the agonist, subsequent agonist effects will not be inhibited by this antagonist. Unfortunately, the P2X3R-responsivity could not be measured immediately after S_3_ because of desensitization. Thus, this protocol can be used only for slowly dissociating antagonists that stick to the receptor as long as the recovery lasts. The comparison of agonist effects at S_4_ and S_7_ sheds light on the fact whether the occupation of the binding site with an agonist protects the receptor from the influence of an antagonist.

## Results

Three structurally diverse P2X3 antagonists, TNP-ATP, A317491, and PPADS (Figure 1, insets) were investigated in the present experiments. It was found that our model describes reasonably well the α,β-meATP-induced current amplitudes and their shapes in the presence of these antagonists or after their wash-out, in the steady state protocol, the wash-out protocol and the dynamic application protocol. The agonist test concentration was kept stable at 10 µM for the wt hP2X3R and its mutants F174A and F301A, because we found previously that this concentration roughly equals the respective EC_50_ values of α,β-meATP in the same expression system [16,17]. In the case of K65A, R281A and N279A, the test concentration of α,β-meATP had to be increased to 100-300 µM in order to cope with the considerably lower activity of this ATP analogue at the receptor mutants. The antagonist concentrations used in interaction with the agonists were gradually increased to a maximum causing nearly complete inhibition. 

The P2X1,3 specific antagonist TNP-ATP (also blocking P2X2/3; [19]) is a structural derivative of the native P2X agonist ATP with additional trinitrophenyl-groups connected to the O2’ and O3’ residues of the ribose ring. As a first step, a concentration-response relationship was constructed with TNP-ATP for its inhibitory effect on the α,β-meATP-induced currents by means of the steady-state protocol (Figure 2A, D). In the same series of experiments, the recovery from desensitization was also tested both in the absence and in the presence of increasing TNP-ATP concentrations (0.3-30 nM) applied 20s before the first agonist application for 110s each with 5-min intervals (steady-state protocol). The wash-out protocol indicated a faster dissociation of the antagonist from the wt P2X3R in comparison with that of α,β-meATP (TNP: k_-1_=0.056±6.1*10^-6^ s^-1^ and α,β-meATP: k_-1_=0.006±0 s^-1^) and an accordingly rapid restitution of the original α,β-meATP current amplitudes at a time-scale of seconds (Figure 2C). The dynamic antagonist application protocol documented a rapid wash-in and comparably rapid wash-out of TNP-ATP at a maximal inhibitory concentration of 30 nM (Figure 2B). In this series of experiments, the first application of α,β-meATP caused a larger response than the subsequent ones. After the fourth α,β-meATP application a stable amplitude was reached. This is due to the failure of a complete recovery from desensitization within a 1-min interval. There was a pronounced overshoot after washing out this antagonist at a time-scale of minutes. The concentration-response curves for TNP-ATP at inhibiting α,β-meATP effects on the investigated P2X3R mutants indicated rather similar K_D_ values, with exception of those for K65A and R281A, where they appeared to be considerably larger than for the other mutants investigated (Figure 2D; Table 1). 

**Table 1:** Equilibrium dissociation constants (K_D_) and binding energies (ΔG) of P2X3R antagonists computed by an extended hidden Markov model.

Antagonists	Mutants	K_D_ (nM) ±S.D.	ΔG (-kJ/mol) ±S.D.	n
TNP-ATP	wt	3.53±0.01	47.73±0.01	29
	*K65A*	*170.45±1.13*	*38.23±0.02*	28
	F174A	5.95±0.04	46.45±0.02	19
	N279A	3.24±0.03	47.94±0.02	22
	*R281A*	*34.01±0.26*	*42.18±0.02*	25
	F301A	3.00±0.04	48.13±0.03	22
A317491	wt	69.87±0.29	40.41±0.01	36
	*K65A*	*316.32±3.66*	*36.71±0.03*	16
	F174A	158.13±1.11	38.41±0.02	26
	N279A	243.04±1.78	37.36±0.02	21
	*R281A*	*875.71±8.15*	*34.21±0.02*	12
	F301A	82.49±0.63	40.01±0.02	22
PPADS	wt	454.75±1.05	35.82±0.01	23

The K_D_ and ΔG values of all mutants differed from the respective values of the wt receptor (P<0.01). Similarly, the wt K_D_ and ΔG values for TNP-ATP, A317491, and PPADS also differed from each other (P<0.05). The K_D_ values of TNP-ATP and A317491 at the K65A and R281A mutants (see italics) were much higher than those measured at the wt receptor or the residual mutants. Accordingly the ΔG values were for the two mutants lower than for the wt receptor or the residual mutants (see italics).

The PPADS is included in the Table only for the matter of completeness, but we consider the values shown as meaningless.

Measurements were performed at the wild-type (wt) receptors and its agonist binding site mutants. The number of experiments (n) represents the sum of all measurements performed with the various protocols to determine K_D_ and ΔG.

The good correlation of all fits with the experimental data suggest that TNP-ATP is a competitive, rapidly reversible antagonist of α,β-meATP at wt hP2X3Rs. The binding sites may be identical with those of ATP itself, without the need to assume additional sites occupied by TNP-ATP. The association rate k_1_ was found to be 15.8±2 µM^-1^ s^-1^ and the dissociation rate was 0.056±0.001 s^-1^, which results in a K_D_ of 3.50±0.02 nM and a binding energy of -47.73±0.01 kJ/mol. Currents measured at all tested mutant receptors could be fitted with our model. The numerical results are summarized in Table 1. The calculated K_D_ values for TNP-ATP were nearly identical at the wt receptor and its mutants F174A, N279A and F301A, but were markedly increased at K65A and R281A suggesting a particular significance of these latter AAs for the binding of this antagonist. These data are congruent with the comparable findings obtained with TNP-ATP as an antagonist.

A317491 has no structural similarity to any of the P2X agonists, but is a specific antagonist for the P2X3R (as well as for P2X2/3; [20]). The steady state protocol allowed on the one hand to determine A317491 (0.03-3 µM) concentration-response curves for its inhibitory action on α,β-meATP currents both at the wt P2X3R and its binding site mutants (Figure 3A, D), and on the other hand the measurement of the recovery from desensitization either in the absence or in the presence of increasing concentrations of A317491 (Figure 3A). Simulated currents could adequately fit experimental current amplitudes and kinetics. A317491 at a concentration (3 µM) which almost abolished the effect of α,β-meATP (10 µM) rapidly dissociated from the wt receptor, immediately after washing it out (Figure 3C). In Figure 3C the amplitudes of the α,β-meATP-induced currents were fitted perfectly well during a wash-out protocol, however, the visible onset of desensitization in the simulations in the continuous presence of the agonist was slightly divergent between the experiments and the fits. The dynamic antagonist application protocol documented a rapid wash-in and comparably rapid wash-out of A317491 at a maximal inhibitory concentration of 3 µM and a marked overshoot after washing out the antagonist (Figure 3B). 

 The concentration-response curves for A317491 in inhibiting α,β-meATP currents at the wt P2X3R and its mutants were similar to those measured for TNP-ATP (compare Figure 2D with Figure 3D). The association rate k_1_ was found to be 6.7±0.02 µM^-1^*s^-1^ and the dissociation rate k_-1_ was 0.47±0.01 s^-1^, which results in a K_D_ of 69.9±0.30 nM, and a binding energy of -40.4±0.01 kJ/mol for the wt P2X3R. The K_D_ values for F174A, N279A and F301A were similar to those measured for the wt receptor, but appeared to increase for the K65A and R281A mutants (P<0.05; Table 1). 

PPADS is a non-selective P2XR antagonist, which has no effect at P2X4Rs and a low efficiency at all other receptor types including P2X1-3 [21,22]. PPADS was reported to block P2XRs in a slowly reversible manner, in contrast to its effects at several P2YR-types, where the recovery after wash-out was fast [22]. The steady-state protocol indicated that increasing PPADS concentrations applied for 5 min each (IC_50_= 0.89±0.61 µM) gradually depressed the amplitude of α,β-meATP (10 µM) currents at the wt P2X3R. Apparently a 5 min superfusion with PPADS is sufficient to reach a maximal inhibitory effect (e.g. for 10 µM PPADS see Figure 4B). Under these conditions k_1_ and k_-1_ values could be determined, and allowed rather convincing fits of P2X3 currents (Figure 4A, C). However, these rate constants proved to be meaningless, because PPADS practically did not dissociate from the receptor after its wash-out, as documented by the dynamic application protocol (Figure 4B). Moreover, the blockade of α,β-meATP (10 µM)-induced currents by PPADS (10 µM) at wt P2X3Rs reached a maximum only very slowly at about 3 min after starting antagonist application (Figure 4B). The agreement between the data points measured experimentally and the corresponding fits were also incomplete in this situation. In consequence, we did not construct concentration-response curves for PPADS at the binding site mutants of wt P2X3Rs.

Because of the slow reversibility of the PPADS-induced blockade of α,β-meATP effects, there was no reason to evaluate the data by a wash-out protocol. Instead, we introduced a protection protocol to find out, whether the agonist and its antagonist occupy the same binding sites at least at an early phase of their inhibitory interaction. This expectation seemed to be valid, because when immediately after washing out the test concentration of α,β-meATP (10 µM), PPADS (400 µM) was applied for 5 s, there was no inhibition of the subsequent α,β-meATP current. However, when PPADS was applied without a preceding agonist superfusion, the subsequent effect of α,β-meATP was markedly depressed (Figure 4C, D). We conclude that the dissociation of the agonist receptor complex prevented subsequent antagonist binding to the receptor. In conclusion, the (pseudo)irreversible blockade of the wt P2X3R by PPADS does not allow the application of a Markov model to describe the relevant receptor functions.

We used throughout the P2X1,3R-selective structural analogue of ATP, α,β-meATP, rather than ATP itself as an agonist. An imminent question is, whether the type of agonist has any influence on the binding energy of the antagonists. In order to answer this question, we performed an additional series of experiments, using ATP and TNP-ATP in our steady state protocol (for the concentration-response of ATP see Figure S1A). We calculated the association and dissociation rates, the K_D_ and the binding energy by using the Markov fit and compared these values with those we obtained from our original experiments using α,β-meATP as an agonist. Using the agonist ATP the binding energy of TNP-ATP was found to be -49.1±0.005 kJ/mol which is within the same range as the one gained of the α,β-meATP experiments (-47.73±0.01 kJ/mol).

## Discussion

**Figure 5:**
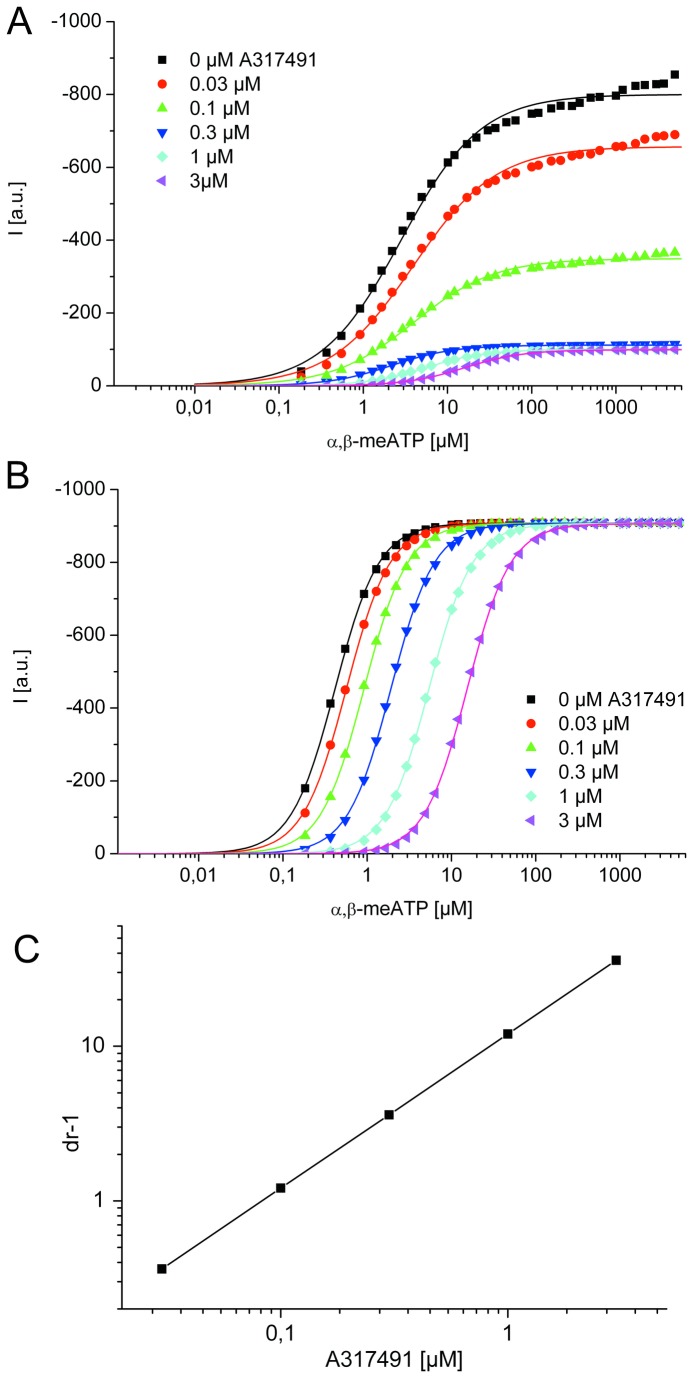
Illustration of the influence of P2X3R desensitization on the Schild-analysis of agonist effects. Concentration-response curves of α,β-meATP in the presence and absence of increasing A317491 concentrations were simulated by the wt P2X3 model (A) and with the same model without desensitization (B). The symbols represent the simulated data points and the lines the corresponding hill fits. A, High agonist concentrations did not induce maximal current amplitudes in the presence of the antagonist. This is due to the fast receptor desensitization which suppresses the current before equilibrium between the agonist and its antagonist is reached at the binding site. The decreased maxima and the non-parallel displacement of the agonist concentration-response curves suggest non-competitive antagonism. B, After setting the desensitization rates (d1-d4) to zero, the competitive character of the model is unmasked. C, The Schild-plot (inset) shows the expected straight line. I (a.u.), current in arbitrary units.

It is difficult to compare results obtained by different research groups with respect to P2X3 antagonists, because they have not been systematically compared in the same preparation and because inadequate experimental protocols, e.g. preincubation times with antagonists not sufficient to reach steady-state conditions, were used [15]. Furthermore, it is not possible to decide by a classic analysis of agonist-antagonist interaction (e.g. Schild plot) whether α,β-meATP and its antagonists interact in a competitive or non-competitive manner at the rapidly desensitizing P2X3R (for P2X1 see 23). The interaction between an agonist and its antagonist is not a simple displacement under equilibrium conditions, but it is complicated by desensitization, because not only the peak current amplitude, but also the current kinetics are altered in the presence of the antagonist. This results in a non-parallel shift of the concentration-response curves for α,β-meATP, and a marked depression of the peak current amplitude (Figure 5A) and may lead to the false conclusion that TNP-ATP acts in a non-competitive manner [19]. By contrast, simulation of the curves without desensitization (by setting the desensitization rates to zero) results in parallel shifts to the right with no change in the respective maxima, allowing the proper determination of the p*A*2 value (Figure 5B)

Therefore, in our Markov model for competitive antagonism there is no direct influence of the antagonists on the recovery rates of the receptor. Nevertheless, there are changes in the observed currents: in the steady state protocol the amount of recovered receptors appears to rise in the presence of increasing antagonist concentrations, when the first application of the agonist is compared to the third one after 60s. This is simulated perfectly well by the model (see Figure 3A; Figure S1B). The overshoot can be explained by the protection of the receptor against agonist-induced desensitization by the bound antagonist. If the antagonist dissociates from the receptor rapidly, there is no additional recovery time and many functional channels are immediately available.

In order to evade the above mentioned limitations, the slowly desensitizing P2X2/3 or chimeric P2X2-3Rs were used previously to obtain reliable results (see Introduction). In fact, TNP-ATP was reported to be an insurmountable, non-competitive antagonist at P2X3 [19], whereas it proved to be a competitive antagonist at both P2X2/3 [15] and P2X2-3 [14,24]. It was concluded that because of the slow off-kinetics of TNP-ATP from the homomeric P2X3R, measurements cannot be (and were not; [19]) carried out in the steady-state condition [24]. 

In addition, there is only a limited amount of data available on the binding of antagonists such as PPADS, which were described to be slowly reversible from P2X2Rs due to the formation of a Schiff base with a K246 [25]; (the analogous AA K223 in P2X3 is outside of the binding pouch). The mutation of Lys to Glu (K246Q) at this position resulted in a rapid reversibility of the PPADS-induced inhibition of P2X2 after wash-out. In analogy, it was concluded that the recovery of P2X2/3 from PPADS inhibition occurred in two steps, one slowly reversible and the other one irreversible [15]. It was also shown that at the Cys-mutants at K68 and K70 of the rapidly desensitizing P2X1R (homologous to K63 and K65 of P2X3), the effect of PPADS did not change in comparison with the wt receptor, although the agonistic ATP effects were inhibited to variable extents [26]. Thus, ATP and PPADS were suggested not to occupy the same AA moieties in the agonist binding pouch (see 27).

In the present study we solved these problems by checking with four different experimental protocols at hP2X3Rs the validity of an extended Markov model to determine K_D_ values and binding energies for the antagonists examined (TNP-ATP, A317491, and PPADS). It was concluded that the reversible antagonists TNP-ATP and A317491 acted in a manner congruent with competitive antagonism. In the case of the (pseudo)irreversible antagonists PPADS [28], this analysis was found to be meaningless. Although our Markov model perfectly described changes observed with the steady-state and wash-out protocols, it failed to supply good fits for the onset and offset of the blockade during the dynamic antagonist application protocol. The fit of the PPADS-induced inhibition was slower and its recovery after antagonist wash-out was faster than in case of the electrophysiologically measured α,β-meATP amplitudes. Because, at least during the early phase of the blockade, the binding of the antagonists could be prevented by agonist application (see the respective protection protocols), we suggest in agreement with others, that the (pseudo)irreversibility of the blockade and the existence of possible accessory binding sites are responsible for the difference between the experimental data and their fits. 

In the case of TNP-ATP, simple logics also suggest a competition between ATP (or its structural analogue α,β-meATP) and the structurally related TNP-ATP. However, A317491 is a tricarboxylic acid structurally unrelated to ATP, which blocks P2X2-3 competitively with a more than two orders of magnitude higher selectivity to P2X3 over P2X1 [14,22]. A317491 was investigated also at the homomeric P2X3R, but increasing concentrations of the antagonist led to a displacement of the agonist and a right shift of the concentration-response curves in a slightly non-parallel manner, although the amplitude of the maximum current did not change (Figure 1 of [20]). Under these conditions a Schild analysis is not really admissible.

All these complications with respect to measurements at homomeric P2X3Rs could be circumvented by our approach. The arguments for this suggestion are the following: (1) The K_D_ values of TNP-ATP and A317491 (3.5 nM and 69.9 nM, respectively) are in the same range as those determined for P2X2-3 by e.g. Neelands et al. [14] (2.2 and 52.1 nM, respectively). (2) The K_D_ values did not depend on the agonist concentration. Whereas at wt P2X3 we used 10 µM α,β-meATP, at the mutant N279A 100 µM α,β-meATP was applied, because of a lower potency of the agonist [17]. Nevertheless, the K_D_ values remained unchanged (Table 1) (3). Two of the investigated AAs (K65A and R281A) AA within the agonist binding site had a critical significance for both agonist (α,β-meATP; [16]) and antagonist binding (TNP-ATP, A317491; present study). 

A survey of the literature indicates a growing interest in studying the mechanism of antagonist binding at P2XRs. Knowledge on the AA composition of the agonist binding pouch of P2XRs was derived for many years from mutagenesis studies [6,29]. The crystallization of the zebrafish P2X4R at first in its closed and then in its ATP-complexed (possibly open) state gave a major thrive to these investigations [27,30]. Whereas originally only the AA residues with significance for agonist binding were studied for these receptors, more recently also AAs involved in antagonist binding have been increasingly investigated [30]. The chimera replacing the region between the third and fourth conserved cysteine residues of the P2X1R with the corresponding part of P2X2 reduced NF449 sensitivity a thousand fold at the P2X1-2R-chimera to that of the P2X1R [31]. This chimera was also involved in determining sensitivity to the antagonist suramin. Structural determinants for the binding of the nanomolar-potent competitive P2X2R antagonist NF770 were clarified with a combined mutagenesis and *in silico* study [32]. In the case of the human P2X7R, F95 has been shown to be important for antagonism/allosteric modulation by a range of species selective antagonists [33,34].

The role of these AAs for antagonist binding to P2X1Rs were investigated without taking into account the rapid desensitization occurring during agonist application [26,31]. We used a kinetic model for agonist binding which was based on the refinement of the original cyclic model for P2X3R operation described by Sokolova et al. [35]. We added a further step to the model, assuming that both diliganded and triliganded receptors could open upon agonist exposure [36]. This correction resulted in better fits of the P2X3 current traces [16]. Eventually, in the present study, we extended the model to fit also agonist-antagonist interactions at P2X3Rs. Since our purpose was to acquire knowledge about the nature of this interaction and the AAs involved, various antagonists were used in combination with various mutants of the P2X3R.

In conclusion, we developed a kinetic model of agonist-antagonist interaction at the rapidly desensitizing P2X3R by identifying individual steps in the transition of this receptor between the closed, open and desensitized states during agonist binding to both antagonist-unbound and antagonist-bound receptors. By means of this model it is possible to perfectly compensate for desensitization induced perturbations of the classic models (e.g. Schild analysis) used to determine equilibrium dissociation constants of agonists.

## Supporting Information

Table S1 **Parameters of the WT P2X3R Markov model (see Fig. 1) for α,β-meATP as agonist and TNP-ATP and A314791 as antagonists.**
(PDF)

Figure S1 **Concentration-dependent inhibition of the ATP-induced current by TNP-ATP (A) and recovery of the α,β-meATP-induced current in the presence of increasing concentrations of A317491 (B).** A, Concentration-response curves for the wt P2X3R simulated by the Markov model (line) to fit the experimentally determined mean current amplitudes (symbols) without and with increasing concentrations of TNP-ATP (0.1 nM - 30 nM) in the superfusion medium. Mean±S.E.M. of 6 experiments. B, Amount of activatable receptors 60 s after first agonist application as a function of antagonist; data derived from steady-state protocol. For experimental details see Fig, 1A.
(TIF)
