# Supplementary material for: Agonist Antagonist Interactions at the Rapidly Desensitizing P2X3 Receptor
Source: PLoS One. 2013 Nov 1;8(11):e79213. doi: 10.1371/journal.pone.0079213 (PMC3815160; doi:10.1371/journal.pone.0079213)
Supplement: Table S1 — Parameters of the WT P2X3R Markov model (see Fig. 1) for α,β-meATP as agonist and TNP-ATP and A314791 as antagonists. (PDF) [file pone.0079213.s001.pdf]

Suppl. Table 1.

Parameters of the WT P2X3R Markov model (see Fig. 1) for α,β-meATP as agonist and TNP-ATP and A314791 as antagonists

			TNP			A317491		
**Rate constants**	**from**	**to**	**k**	**std**	**equilibrium**	**k**	**std**	**equilibrium**
$3a_1$	1	2	5.76	0	1,582	5.76	0	1.582
a_{-1}	2	1	3.64	0	0,6319	3.64	0	0.6319
$2a_1$	2	3	3.84	0	0,5275	3.84	0	0.5275
$2a_{-1}$	3	2	7.28	0	1,896	7.28	0	1.896
a_1	3	4	1.92	0	0,1758	1.92	0	0.1758
$3a_{-1}$	4	3	10.92	0	5,687	10.92	0	5.687
$3k_1$	1	5	0.04751	4.68E-06	0,6358	20.06	0.0582	42.94
**k_{-1}**	**5**	**1**	**0.05597**	**6.10E-06**	**1,573**	**0.4672**	**0.001365**	**0.02329**
$2k_1$	5	8	0.03167	3.12E-06	0,2119	13.37	0.0388	14.31
$2k_{-1}$	8	5	0.1119	1.22E-05	4,718	0.9344	0.00273	0.06987
$2k_1$	2	6	0.03167	3.12E-06	0,2119	13.37	0.0388	28.62
k_{-1}	6	2	0.05597	6.10E-06	4,718	0.4672	0.001365	0.03494
**k_1**	**3**	**7**	**0.01584**	**1.56E-06**	**0,07064**	**6.687**	**0.0194**	**14.31**
k_{-1}	7	3	0.05597	6.10E-06	14,16	0.4672	0.001365	0.06987
$2a_1$	5	6	3.84	0	0,5275	3.84	0	1.055
a_{-1}	6	5	3.64	0	1,896	3.64	0	0.9479
a_1	6	7	1.92	0	0,1758	1.92	0	0.2637
$2a_{-1}$	7	6	7.28	0	5,687	7.28	0	3.792
a_1	8	9	1.92	0	0,1758	1.92	0	0.5275
a_{-1}	9	8	3.64	0	5,687	3.64	0	1.896
k_1	6	9	0.01584	1.56E-06	0,07064	6.687	0.0194	7.156
$2k_{-1}$	9	6	0.1119	1.22E-05	14,16	0.9344	0.00273	0.1397
k_1	8	10	0.01584	1.56E-06	0,07064	6.687	0.0194	4.771
$3k_{-1}$	10	8	0.1679	1.83E-05	14,16	1.402	0.004095	0.2096
d_{-1}	11	1	273.4	0	713,9	273.4	0	713.9
d_1	1	11	0.3829	0	0,001401	0.3829	0	0.001401
d_{-2}	12	2	8.1	0	1,247	8.1	0	1.247
d_2	2	12	6.498	0	0,8022	6.498	0	0.8022
d_{-3}	13	3	0.03762	0	0,002175	0.03762	0	0.002175
d_3	3	13	17.3	0	459,8	17.3	0	459.8
d_{-4}	14	4	8.28E-05	0	3,80E-06	8.28E-05	0	3.80E-06
d_4	4	14	21.81	0	2,63E+05	21.81	0	2.63E+05
$3a_1$	11	12	5.76	0	906,8	5.76	0	906.8
$1a_{-1}$	12	11	0.006352	0	0,001103	0.006352	0	0.001103
$2a_1$	12	13	3.84	0	302,3	3.84	0	302.3
$2a_{-1}$	13	12	0.0127	0	0,003308	0.0127	0	0.003308
a_1	13	14	1.92	0	100,8	1.92	0	100.8
$3a_{-1}$	14	13	0.01906	0	0,009925	0.01906	0	0.009925
d_{-1}	15	5	273.4	0	713,9	273.4	0	713.9
d_1	5	15	0.3829	0	0,001401	0.3829	0	0.001401
d_{-2}	16	6	8.1	0	1,247	8.1	0	1.247
d_2	6	16	6.498	0	0,8022	6.498	0	0.8022
d_{-3}	17	7	0.03762	0	0,002175	0.03762	0	0.002175
d_3	7	17	17.3	0	459,8	17.3	0	459.8
$2a_1$	15	16	3.84	0	302,3	3.84	0	604.5
a_{-1}	16	15	0.006352	0	0,003308	0.006352	0	0.001654
a_1	16	17	1.92	0	100,8	1.92	0	151.1
$2a_{-1}$	17	16	0.0127	0	0,009925	0.0127	0	0.006617
$3k_1$	11	15	0.04751	4.68E-06	0,6358	20.06	0.0582	42.94
$k-_1$	15	11	0.05597	6.10E-06	1,573	0.4672	0.001365	0.02329
$2k_1$	12	16	0.03167	3.12E-06	0,2119	13.37	0.0388	28.62

			TNP			A317491		
**Rate constants**	**from**	**to**	**k**	**std**	**equilibrium**	**k**	**std**	**equilibrium**
k_{-1}	16	12	0.05597	6.10E-06	4,718	0.4672	0.001365	0.03493
k_1	13	17	0.01584	1.56E-06	0,07064	6.687	0.0194	14.31
k_{-1}	17	13	0.05597	6.10E-06	14,16	0.4672	0.001365	0.06987
a_1	18	19	1.92	0	100,8	1.92	0	302.3
a_{-1}	19	18	0.006352	0	0,009925	0.006352	0	0.003308
d_{-2}	19	9	8.1	0	1,247	8.1	0	1.247
d_2	9	19	6.498	0	0,8022	6.498	0	0.8022
d-$_1$	18	8	273.4	0	713,9	273.4	0	713.9
d_1	8	18	0.3829	0	0,001401	0.3829	0	0.001401
$2k_1$	15	18	0.03167	3.12E-06	0,2119	13.37	0.0388	14.31
$2k_{-1}$	18	15	0.1119	1.22E-05	4,718	0.9344	0.00273	0.06987
k_1	16	19	0.01584	1.56E-06	0,07064	6.687	0.0194	7.156
2 k_{-1}	19	16	0.1119	1.22E-05	14,16	0.9344	0.00273	0.1397
d_{-1}	20	10	273.4	0	713,9	273.4	0	713.9
d_1	10	20	0.3829	0	0,001401	0.3829	0	0.001401
k_1	18	20	0.01584	1.56E-06	0,07064	6.687	0.0194	4.771
$3k_{-1}$	20	18	0.1679	1.83E-05	14,16	1.402	0.004095	0.2096
o_1	7	23	1.00E+04	0	10	1.00E+04	0	10
o_{-1}	23	7	1000	0	0,1	1000	0	0.1
k_{-1}	23	21	0.05597	6.10E-06	14,18	0.4672	0.001365	0.07006
k_1	21	23	0.01579	1.56E-06	0,07051	6.669	0.01935	14.27
o_{-1}	21	3	1000	0	0,1	1000	0	0.1
o_1	3	21	1.00E+04	0	10	1.00E+04	0	10
o_1	4	22	1.00E+04	0	10	1.00E+04	0	10
o_{-1}	22	4	1000	0	0,1	1000	0	0.1
$3a_{-1}$	22	21	10.92	0	5,688	10.92	0	5.688
a_1	21	22	1.92	0	0,1758	1.92	0	0.1758

Constraints:

ScaleRate: 1 2 2 3
ScaleRate: 1 2 3 4
ScaleRate: 1 2 21 22
ScaleRate: 1 2 11 12
ScaleRate: 1 2 12 13
ScaleRate: 1 2 13 14
ScaleRate: 2 1 3 2
ScaleRate: 2 1 4 3
ScaleRate: 2 1 22 21
ScaleRate: 12 11 13 12
ScaleRate: 12 11 14 13
ScaleRate: 1 11 2 12
ScaleRate: 1 11 3 13
ScaleRate: 1 11 4 14
ScaleRate: 11 1 12 2
ScaleRate: 11 1 13 3
ScaleRate: 11 1 14 4
ScaleRate: 1 2 5 6
ScaleRate: 1 5 2 6
ScaleRate: 1 5 3 7
ScaleRate: 1 5 11 15
ScaleRate: 1 5 12 16
ScaleRate: 1 5 13 17
ScaleRate: 5 1 6 2
ScaleRate: 5 1 7 3
ScaleRate: 5 1 15 11
ScaleRate: 5 1 16 12

ScaleRate: 5 1 17 13
ScaleRate: 2 1 6 5
ScaleRate: 1 11 5 15
ScaleRate: 11 1 15 5
ScaleRate: 12 11 16 15
ScaleRate: 5 6 6 7
ScaleRate: 5 6 15 16
ScaleRate: 5 6 16 17
ScaleRate: 6 5 7 6
ScaleRate: 16 15 17 16
ScaleRate: 5 15 6 16
ScaleRate: 5 15 7 17
ScaleRate: 15 5 16 6
ScaleRate: 15 5 17 7
ScaleRate: 1 2 8 9
ScaleRate: 2 1 9 8
ScaleRate: 1 5 5 8
ScaleRate: 1 5 6 9
ScaleRate: 1 5 15 18
ScaleRate: 1 5 16 19
ScaleRate: 1 11 8 18
ScaleRate: 11 1 18 8
ScaleRate: 5 1 8 5
ScaleRate: 5 1 9 6
ScaleRate: 5 1 18 15
ScaleRate: 5 1 19 16
ScaleRate: 12 11 19 18
ScaleRate: 8 9 18 19
ScaleRate: 8 18 9 19
ScaleRate: 18 8 19 9
ScaleRate: 1 11 10 20
ScaleRate: 11 1 20 10
ScaleRate: 1 5 8 10
ScaleRate: 1 5 18 20
ScaleRate: 5 1 10 8
ScaleRate: 5 1 20 18
FixRate: 3 21
FixRate: 21 3
FixRate: 7 23
ScaleRate: 1 5 21 23
ScaleRate: 5 1 23 21
FixRate: 23 7
FixRate: 4 22
FixRate: 22 4
LoopBal: 3 2 12 13 14 4
FixRate: 1 2
FixRate: 2 1
FixRate: 1 11
FixRate: 11 1
